# The Effects of Ranitidine and Hydrocortisone on the Complications of Femoral Artery Obstruction Treated by Streptokinase Following Cardiac Catheterization in Pediatric Patients with Congenital Heart Diseases

**DOI:** 10.5812/ircmj.7248

**Published:** 2013-02-05

**Authors:** Noormohammad Noori, Ghasem Miri Aliabad, Mehdi Mohammadi, Maziar Mahjoubifard, Alireza Jahangiri Fard

**Affiliations:** 1 Research Center for Children and Adolescent Health, ALI-EBN E-ABITALEB Hospital, Zahedan University of medical sciences, Zahedan, IR Iran; 2 Fellowship of Cardiac Anesthesia, Rajaie Cardiovascular Medical & Research Center, Tehran University of Medical Sciences, Tehran, IR Iran

**Keywords:** Catheterization, Thrombosis, Streptokinase, Child

## Abstract

**Background:**

The most important complication following cardiac catheterization required urgent therapeutic management is vessel obstruction and arterial thrombosis. The morbidity following this complication can be decreased by surgery intervention and/or thrombolytic drugs.

**Objectives:**

In this study we evaluated the effects of ranitidine and hydrocortisone on pediatric patients with congenital heart diseases who suffered from femoral artery obstruction following cardiac catheterization on decreasing the events after streptokinase administration.

**Materials and Methods:**

This semi experimental study was conducted on 47 patients among 600 cases who underwent cardiac catheterization from April 2002 to December 2011.The patients suffered from distal vessel obstruction following cardiac catheterization with no response to surgery intervention, were enrolled and divided in two groups. Streptokinase was administrated in both groups. Patients in group 2 (25 cases), received ranitidine and hydrocortisone before streptokinase administration. In group 1 (22 cases), the loading dose of streptokinase was 2000IU/kg/ in 20-30 minutes/ infusion and thereafter streptokinase was administrated 1000 IU /kg/hour. In group 2, the loading dose was 3000IU/kg in 20-30 minutes /infusion and 1500 IU/kg/hour as maintenance dose. The infusion dose of streptokinase was decreased and then terminated in 2-3 hours by the time arterial pulse was detected by pulse oximetry.

**Results:**

There were 13 (59, 1%) male and 9 (40.9%) female patients in group 1. In group 2, there were 15 (60%) male and 10 (40%) female cases (P = 0.949). Patients in both groups were matched well regarding age, body weight, height and the duration of streptokinase infusion (P < 0.05). The incidence of hematoma was higher in group 1 than group 2 (P = 0.032). the patients of Group 1 required more blood transfusion than group 2 because the incidence of bleeding was more in the first group (P = 0.042). 12 patients in group 1 required fresh frozen plasma transfusion versus 4 patients in group 2 (P = 0.049). Local oozing was detected more in group 1 (P = 0.042). Significant bleeding was occurred in 6 cases in group 1; however this event did not occurrin any patients in group 2 (P = 0.007). Although 4 patients in group 1 suffered from anaphylactic shock after streptokinase administration but no patients in group 2 did. (P = 0.041).

**Conclusions:**

Based on the results of this study, we concluded that streptokinase was able to remove the vessel thrombosis at the site of cardiac catheterization and ranitidine and hydrocortisone administration before streptokinase may be effective in order to reduce the complications related to thrombolytic drugs; however the experience of the performer is an issue of concern in this matter.

## 1. Background

Cardiac catheterization is one the most important techniques in diagnosing the congenital heart diseases(1,2). In recent decade, not only cardiac catheterization in pediatric patients has been performed as a diagnostic instrument to evaluate the anatomy of the heart and its hemodynamic condition, but also its therapeutic role has caused an evaluation in pediatric cardiology specialty(1,3).Cardiac catheterization technique may lead to some complications. The most important events are: cardiac arrhythmia, bleeding,tamponade, cardiac rupture, death and femoral artery thrombosis which the latter is reported inabout9% of the patients(1,2). The most common reason for femoral artery thrombosis in pediatrics is because of the catheter in cardiac catheterization which is reported in 8 to 39% of the cases with body weight (BW) less than 14 kg in spite of prophylacticheparinization(3-5). The signs and symptoms of this complication are the lack of arterial pulse, pale skin and leg paralysis(4,6). Femoral artery thrombosis is mostly observed after cardiac catheterization and in extreme and un-treated cases this complication may lead to gangrene(6,7). In the study ofVeldmanet al. the incidence of thromboembolic event was reported 5.1 in 100,000 live births. Neonatal arterial ischemic stroke occurred in large carotid vessels in 2/3 of the cases. In patients with congenital heart disease (CHD) the incidence of venousthromboemboliwas reported 50% and the incidence of arterial thromboembolic events were observed in 70% of the patients with age of less than 6 months(8).Raffiniand colleagues concluded in their study that thrombolytic therapy has been increased in children in the past two decades and that arterial thrombosis following catheterization had been reported the most common indication for thrombolytic drugs administration(9).Gittinset al. showed in their study thatalteplaseadministration was more effective in order to prevent clot formation in central catheters in hemodialysis than heparin which resulted into less morbidity(10). In the study ofFilisand colleagues the most common complication after catheterization in adults was femoral artery injury(11).Monagleet al. recommended in their study that the level of fibrinogen, thrombin clotting time (TCT),prothrombintime (PT) and activated partialthromboplastintime (aPTT) should be evaluated in time of treatment with streptokinase(12). The treatment of this complication is based on medical treatment. First the catheter must be extracted(6,7)and if there is no pulse in following four hours the thrombolytic drugs may be administrated including streptokinase,urokinaseandalteplase(12,13). Intravenous streptokinase is an effective and safe treatment for femoral artery thrombosis following cardiac catheterization in children(9,12,14).

## 2. Objective

Regarding the fact that cardiac catheterization is more required for children recently and due to its complications including thrombosis, in this study we administrated hydrocortisone and ranitidine before streptokinase administration in order to reduce the side effects of the mentioned thrombolytic drug.

## 3. Methods and Materials

Approval of the study protocol was granted by institutional review board and written informed consent was obtained from the parents of the patients. This semi experimental study was conducted on the patients with congenital heart diseases with the indication of cardiac catheterization and following artery injury at heart clinic in AliAsgharhospital,Zahedan, Iran from April 2002 to December 2011. The catheterization was conducted by one pediatric cardiologist and GE instrument for all the cases. 600 patients with mean age of 2-180 months who underwent cardiac catheterization were enrolled in this study. Among a total, 47 (7.83%) cases suffered from vessel complications. The diagnosis of the events was based on observation (change of color of the leg: pale skin), examination (change of temperature of the leg and noevidence of distal pulse), pulse-oximetryand Doppler echocardiography (8 patients).The patients were divided in 2 groups. Group 1: 22 patients are the cases who had no arterial pulse, with cold and pale leg following cardiac catheterization with no response to basic management within 3-4 hours. For three patients in this group, surgery consult was ordered with a heart surgeon who refused to remove the clot with the aid ofForgatycatheter and therefore streptokinase administration became necessary in order to prevent gangrene formation. Two third (2/3) of the cases in group 1 were heparinized before streptokinase administration and due to no response to heparin, streptokinase (BiotecHeber, Cuba, No 1382) was administrated. The 250,000 I.U vial of streptokinase was solubilized by normal saline (N/S) 0.9% solution (5 cc) and then the anticoagulant was diluted with 45 cc of N/S. From the prepared solution, 2000 I.U /Kg were injected as bolus/I.V in 20-30 minutes, thereafter the infusion of streptokinase: 1000 I.U /kg/hour was administrated by pomp for the cases. By the time arterial pulse was detected and normal color and temperature of the leg were observed,the dosage of streptokinase was decreased to minimal dose and the infusion was terminated after 2-3 hours. The results of the treatment were followed up by observation (normal color of the leg), examination (normal temperature of the leg and distal pulse detection), pulseoximetryand Doppler echocardiography (8 patients). The patients in group 2: 25 cases received 1 dose of hydrocortisone and 1 dose of ranitidine before receiving streptokinase. The solution of streptokinase was prepared in the same way and from the prepared solution, 3000 I.U /Kg was injected as bolus/I.V in 20-30 minutes ,afterwards the infusion of streptokinase :1500 I.U /kg/hour was administrated by a pomp for the patients. The dosage of streptokinase was decreased to minimal dose when arterial pulse was detected and normal color and temperature of the leg were observed and the infusion was terminated after 2-3 hours. The demographic data of the patients including: gender, age, bodyweight,heightandlaboratory results including: PT, PTT and hemoglobin (Hb) and streptokinase side effects were noted in the designed forms for both groups. The patients were also divided in 2 groups based on their cardiac disease: simple (PAVSD, ASD, VSD, PDA) and complex (TF, TAPVC, TGA, CAVSD, and DORV). In both groups, the infusion of streptokinase was terminated in case of massive bleeding and hematoma following streptokinase administration. In order to prevent gastrointestinal (GI) bleeding and/or allergy in group 2 patients, H2 blocker (ranitidine and/or cimetidine) and corticosteroid were injected 1 hour before streptokinase administration. In this study,the relationships between gender, age, weight, the type of heart disease and the duration of streptokinase infusion were analyzed in both groups. The data were analyzed with SPSS software and statistical Chi-square test and T-test.

## 4. Results

In group 1(22cases), there were 13 male (59.1%) and 9 (40.9%) female patients and in group 2(25 cases), 15 patients (60%) were male and 10 cases (40%) were female with no significant differences among the groups (P = 0.949). In this study, two study groups received streptokinase since there was no femoral pulse following catheterization. However,patients ofgroup 2 received hydrocortisone and H2 blocker before streptokinase administration. Both groups were matched regarding age, weight, height, laboratory parameters, and the duration of streptokinase infusion (Tables 1 and 2). Two patients in group 1 and twopatientsin group 2 suffered from arrhythmia with no significant difference. Five patients in group 1 and 8 patients in group 2 received morphine and 17 cases receivedpethidinein each group. Loss of pulse was confirmed in all (100%) patients.Patients ofGroup 1 received heparin during the first 4 hours after femoral thrombosis, however no pulse was detected, and thereafter streptokinase were administrated. In contrast, no patients in group 2 received heparin before streptokinase administration. Twelvecases suffered from hematoma in group 1 (22 cases) versus 6 patients in group 2 (25 patients) after receiving streptokinase with significant difference (P = 0.032). In first group of patients, who received heparin and streptokinase, the incidence of bleeding and anemia was higher (12 cases) than the second group (5 cases). This difference was statistically significant (P = 0.05) which can be due to the positive effects of the drugs administrated before streptokinase. In group 1, 14 patients required blood transfusion versus 8 patients in group 2 with significant difference (P = 0.042). Twelve patients in group 1 and 4 patients in group 2 required fresh frozen plasma (FFP) (P = 0.049). In both groups, femoral pulse was detected after streptokinase administration with no failure.14patients in group 1 and 8 patients in group 2 experienced local oozing (P = 0.042). Significant bleeding was observed in 6 patients in group 1, however no cases in group 2 who had received H2 blocker and hydrocortisone before streptokinase suffered from this event (P = 0.007). This result can be due to the positive effect of H2 blocker and hydrocortisone on preventing the incidence of bleeding after streptokinase administration. In this study, patients with different types of congenital heart diseases (PS,PDA,PAVSD,severeMR, AS, CoA, AVSD, VSD, TGA, TF, TAPVC, TA, and PPH) who required diagnostic catheterization were enrolled. Anaphylactic shock was occurred in 4 patients in group 1, however no patients in group 2 suffered from this complication (P = 0.041). Fever was only detected in one patient in group 1; seizure was also occurred in one patient in this group; however, no patients in group 2 suffered from fever or seizure.

**Table 1:** The Demographic Data of the Patients

Factors	Group		P value
	1	2	
**Age, y, Mean ± SD**	19.05 ± 16.43	17.52 ± 16.47	0.753
**Height, Mean ± SD**	73.13 ± 16.29	72.58 ± 23.77	0.926
**Weight, Mean ± SD**	7.47 ± 3.32	8.62 ± 6.52	0.443

**Table 2:** Laboratory Parameters

Laboratory Parameters	Groups		P value
	1	2	
**SG, Mean ± SD**	1012.36 ± 6.46	1012.64 ± 5.48	0.875
**Na, Mean ± SD**	139.95 ± 3.35	137.96 ± 4.65	0.103
**K, Mean ± SD**	4.63 ± 0.5	4.6 ± 0.48	0826
**PT, Mean ± SD**	13.77 ± 2.67	13.38 ± 0.97	0.491
**PTT, Mean ± SD**	42.5 ± 9.09	42.56 ± 13.38	0.986
**BUN, Mean ± SD**	15.13 ± 5.96	14.39 ± 4.8	0.640
**Cr, Mean ± SD**	0.6 ± 0.18	0.55 ± 0.22	0.475
**Ca, Mean ± SD**	9.02 ± 0.58	8.98 ± 0.64	0.812
**Time Streptokinase, Mean ± SD**	11.95 ± 17.96	9. 8 ± 11.45	0.622

## 5. Discussion

In this study, 47 patients (7.83%) suffered from vessel obstruction at the site of the catheter insertion. This event can be due to the young age of the cases, catheters with small size and lack of access to the proper instruments. In the study of Liu(4)and Kothari(15), the incidence ofvessel obstruction following catheterization was reported the same with this study in spite of prophylacticheparinization. The duration of streptokinase infusion was 4-30 hours (mean: 11.5 hours) in the study of Kothari et al. in 1996(15), which is similar to this study. In the study that was conducted in Holland byBrusF and colleagues at Sophia Children’s Hospital in 1990 on 205 children, 7.3% of the cases suffered from femoral artery thrombosis and in hematologic evaluation, PT level had been increased(16). In our study,the incidence of vessel obstruction was 7.83% and PT level was in the range of 12-25.5 (mean: 13.77 ± 2.3) similar with the mentioned study. Liu(4)and Bulbul(17)concluded in their study that the incidence of vessel obstruction following cardiac catheterization was higher in the patients with body weight less than 10 kg. The authors have not mentioned the range and mean body weight of the cases in their study. In recent study the cases in both groups were matched regarding body weight. The therapeutic effect of streptokinase on vessel obstruction regarding the gender of the patients has not been evaluated in the studies before. Both groups were matched well regarding their gender in our study (P = 0.949), therefore there was no significant correlation between gender and the duration of streptokinase infusion among our cases. In recent study both groups were also matched well regarding their age (P = 0.753). Kothari et al. did not mention this correlation(15); however Liu(4)and Bulbul(17)stated in their studies that vessel obstruction and thromboembolic events may occur more in patients with younger age. In this study, the duration of streptokinase infusion was more than 24 hours in the patients with complex heart disease. Both groups of cases in this study were matched regarding the duration of infusion and the types of cardiac diseases (P = 0.622). There were no evaluations of the therapeutic effect of streptokinase and the duration of streptokinase infusion on thromboembolic events following catheterization regarding the types of the cardiac diseases in the past studies. In this study,arterial pulse was detected in all the patients after streptokinase infusion. In seven patientsofgroup 1 and four cases in group 2 in our study,the arterial pulse was detected after 24 hours, comparing the study of Kothari et al. which in 2 patients of 12 cases,the arterial pulse was detected 24 hours after streptokinase infusion(15)similar totheour study. Local oozing was the most common complication after streptokinase infusion that this event was reported in 14 patients in group 1 and 8 cases in group 2 (46.8%) that was managed in some cases after local compression or termination of streptokinase infusion. In the studies of Kothari(15)in 1996 andMonagle(12,18)in 2008 and 2004,the most common complication was also local oozing, which 2 of 12 casesin the first studyand 6 of 20 casesin the second studysuffered from local oozing similar to our study. The other event after streptokinase administration was hematoma that was detected in 12 patients in group 1 and 6 patients in group 2 (38.3% among total).In4patients (8.5%), anaphylactic shock was occurred. All four cases were in group 1 and the complication was relieved after terminating the streptokinase infusion and with the aid of supportive care; however anaphylactic shock was not occurred in group 2. No complications were reported in 29 patients among total (61.7%), and irreversible events were not occurred in any cases. Patients in group 2 who received H2 blocker and corticosteroid before streptokinase administration and applied elastic bandages by the time of streptokinase administration did not suffer from any life threatening complications except for local oozing and/or mild hematoma. Regarding the fact that cardiac catheterization is more required for children recently and due to its complications including femoral artery thrombosisandbeinginaccessibleto pediatric heart surgeon in order to perform Fogarty to remove the clot, performing other techniques are necessary in order to manage these patients. Medical management and streptokinase administration may be useful in these conditions. In this study,medical management and streptokinase infusion were performedfor these patients and regarding their satisfactory results and no irreversible complications in most of the patients, we concluded that streptokinase may act as the most effective treatment for pediatric patients with femoral artery thrombosis following cardiac catheterization. We also concluded that ranitidine and hydrocortisone administration before streptokinase may be effective in order to decrease the incidence of complications after streptokinase administration; however the experience of the performer is an issue of concern in this matter.
